# Social exclusion at the intersections of immigration, employment, and healthcare policy: A qualitative study of Mexican and Chinese immigrants in California

**DOI:** 10.1016/j.socscimed.2022.114833

**Published:** 2022-02-18

**Authors:** Michelle K. Nakphong, Maria-Elena De Trinidad Young, Brenda Morales, Iris Y. Guzman-Ruiz, Lei Chen, Kathryn G. Kietzman

**Affiliations:** a Department of Community Health Sciences, Fielding School of Public Health, University of California, Los Angeles, Los Angeles, CA, USA; b Center for Health Policy Research, University of California, Los Angeles, Los Angeles, CA, USA; c Department of Public Health, School of Social Sciences, Humanities, and Arts University of California, Merced, Merced, CA, USA; d Department of Social Welfare, Luskin School of Public Affairs, University of California, Los Angeles, Los Angeles, CA, USA

**Keywords:** Emigration and immigration, Health policy, Employment, Insurance coverage, Social inclusion, Social hierarchy

## Abstract

While immigrants in the US suffer poor access to healthcare in general, access within immigrant populations varies notably by legal status and employment. Intersections between immigration, employment, and healthcare policy have shaped immigrants’ access or exclusion from healthcare; however, little research has examined how immigrants experience and navigate these intersections. Drawing on social exclusion theory and the theory of bounded agency, we aimed to investigate Mexican and Chinese immigrants’ experiences of exclusion from healthcare as one key dimension of social exclusion–and how this was shaped by interactions with the institutions of immigration and employment. The examination of two ethnic immigrant groups who live under the same set of policies allows for a focus on the common impacts of policy. We selected Mexican and Chinese immigrants as the two largest subgroups in California’s Latinx and Asian immigrant population. We use a policy lens to analyze qualitative data from the mixed-methods Research on Immigrant Health and State Policy (RIGHTS) Study, involving 60 in-depth interviews with Mexican and Chinese immigrants in California between August 2018–August 2019. We identified two primary themes: pathways of social exclusion and access, and strategies used to address social exclusion. Findings show that immigrants’ exclusion from healthcare is fundamentally linked to legal status and employment, and that immigrants navigate difficult choices between opportunities for improved employment and changes in legal status. We argue that multiple categories of legal status affect immigrants’ employment opportunities and social position, which, in turn, translates to stratified healthcare access. Our findings support the literature establishing legal status as a mechanism of social stratification but challenge legal-illegal binary paradigms.

## 1. Introduction

Latinx and Asian immigrants in the United States (US) are disproportionately uninsured (Alegría et al., 2006). In California in 2020, 23.5% and 9.4% of Latinx (the gender-neutral term for those of Latin-American descent) and Asian noncitizens were uninsured, respectively, compared to 2.7% of US-born Whites (UCLA Center for Health Policy Research, 2020). Latinx and Asian immigrants also have low rates of preventive care utilization, including cancer, hypertension, and cholesterol screenings which likely contribute to higher rates of unaddressed chronic illnesses (Rodríguez et al., 2009; Yao and Hillemeier, 2014). Mexican immigrants are 2.3 times more likely to have undiagnosed diabetes compared to US-born Mexicans (Barcellos et al., 2012). Asian immigrants may be at higher risk for chronic illnesses such as osteoporosis, stomach cancer, and liver cancer, which are exacerbated by higher rates of uninsurance and lower rates of healthcare usage (Clough et al., 2013).

Disparities in healthcare access among immigrants may be attributed to differences in employment opportunities, as most (58.7%) US adults have employer-sponsored insurance (Tolbert et al., 2021). Uninsurance among Latinx and Asian immigrants is driven by their limited access to employer-sponsored insurance (Brown and Yu, 2009; Cook et al., 2014). Employment characteristics largely account for the variation in employer health insurance coverage among immigrants. Immigrants in lower wage jobs are less likely to have employer-based health insurance (Carrasquillo et al., 2000). Legal status also plays a notable role in insurance coverage among employed immigrants (Ponce et al., 2008). Nationally, the gap in employer-sponsored insurance coverage between non-citizens and native-born populations is 14.2%, compared to 3.8% between naturalized citizens and native-born, after adjusting for human capital and employment characteristics (Buchmueller et al., 2007).

The intersection between employment and healthcare access for immigrants is hardly surprising given the historical linkages between healthcare, employment, and immigration policy domains. Rooted in the industrial revolution, health insurance was created to improve workers’ health for industrial efficiency. Efforts to establish government-sponsored universal health care in the US have been unsuccessful, giving rise to employer-sponsored insurance as the dominant form of health insurance (Blumenthal, 2006). Employment and immigration policy have long been closely tied. Immigration policies, such as the 1942 Bracero program, have been relaxed to fill labor shortages, while others, such as the 1882 Chinese Exclusion Act and subsequent Geary Act, were enacted to bar immigrants’ participation in the labor force (Hirschman and Mogford, 2009). Similar policies have continued; the 2017 “Buy American and Hire American” executive order (E.O. 13, 788 of Apr 18, 2017) sought to favor American workers by tightening the H1–B guest worker visa program.

How immigration, employment, and healthcare policy domains intersect to impact the lived experiences of immigrants is under- researched. Specifically, as we describe in this paper, their intersection influences citizenship hierarchies and social location. Most studies of this topic have focused on 1) barriers to healthcare access for specific immigrant groups (e.g., undocumented, types of workers) (Gleeson, 2012; Pourat et al., 2017; Van Natta et al., 2019) or 2) working conditions (i.e., compensation, discrimination) and occupational hazards (de Castro et al., 2006; Montes De Oca et al., 2011). These studies show legal status is a determinant of health that may shape health-related trajectories along the life-course via employment. However, a gap in the literature is how immigrants navigate different legal statuses, including changes (Torres and De Trinidad Young, 2016), and how they are linked to variations in employment and healthcare access over time. This study aims to address this gap by investigating Chinese and Mexican immigrants’ experiences with legal status, employment, and healthcare by capturing their retrospective accounts of individual transitions in each of these policy domains.

In particular, a qualitative examination of two groups with distinct countries of origin and trajectories of incorporation but who make their lives under the same set of policies allows for a focus on how policies intersect across the numerous strata of legal status and immigrant-dominant labor sectors. Exploring Mexican and Chinese immigrants’ experiences highlights the dynamic, intertwined processes of social structures and interactions in work, immigration, and healthcare spheres to produce disadvantage. In other words, while these two groups experience *migration* differently, their experiences of the intersections of policies can provide critical insights into the ultimate impacts of policy on immigrants.

Our study elaborates on immigrants’ trajectories related to the intersections of immigration, employment, and healthcare policy and what this means for their healthcare access. Examining multiple social institutions in an investigation of immigrants’ work and health has the potential to inform the adoption of effective policy interventions (Fujishiro et al., 2021). By examining immigrants’ lived experiences, we also privilege the voices of immigrants and how they make meaning of their realities (Hesse-Biber, 2010).

## 2. Conceptual framework

Our conceptual framework is informed by two theories: Kabeer’s (2000) framework of social exclusion and the theory of bounded agency (Evans, 2007; Schoon and Heckhausen, 2019). Social inclusion is defined as “the process of improving the terms for individuals and groups to participate in society” as well as “the process of improving the ability, opportunity, and dignity of people, disadvantaged on the basis of their identity, to take part in society” (World Bank, 2013, p. 50). Social exclusion theory (Kabeer, 2000) provides a social policy lens to qualitatively examine the institutional mechanisms that produce exclusion and access. Multiple institutions (e.g., government, organizations, communities) and the access they provide intersect and overlap, such that “access and exclusion in one institutional domain can be offset or exacerbated by access and exclusion in another (Kabeer, 2000, p. 87).” These intersections produce structures of opportunity and access to resources that shape experiences of social inclusion or exclusion (whole or partial exclusion from full participation in society), resulting in the segmentation of society.

For example, US immigration laws have been used to racialize Chinese and Mexicans’ legal status, reinforcing social hierarchies related to race/ethnicity, country of origin, gender, and class among newly arrived immigrant groups, ultimately influencing their integration into society (Ngai, 2004; Viruell-Fuentes et al., 2012). Throughout US history, immigration policies have reinforced an “axis of stratification” between citizens and noncitizens that has closely aligned with national attitudes and priorities related to racial exclusion (Menjívar and Abrego, 2012). For example, by the 1920s, immigration laws imposed quotas that restricted immigrants from Southern and Eastern-European countries, completely excluded Asian migration or naturalization, and instituted border policing to control Mexican migrants (Ngai, 2004). Immigration-related policies reproduce inequalities through the unequal distribution of resources and opportunities based on citizenship status at the federal, state, and local levels (Kim, 2018; Menjívar and Abrego, 2012). Thus, legal status also structures immigrants’ experiences and legitimizes their symbolic denigration.

This can be seen in how federal-level immigration policies have extended their reach over employment and healthcare policies. The 1986 Immigration and Reform and Control Act (IRCA) marked a significant point in immigration policy establishing penalties for employers who hired undocumented workers. The 1996 Illegal Immigration Reform and Immigrant Responsibility Act (IIRIRA) further created employment verification programs (i.e., “E-Verify”) to help employers comply with federal law (Feller, 2009). The 1996 Personal Responsibility and Work Opportunity Reconciliation Act (PRWORA) also enacted changes in the welfare program, including limits for “qualified aliens” on participation of means-tested aid programs including a five-year waiting period for Medicaid benefits for lawful permanent residents (LPRs) (Kaushal and Kaestner, 2005). These policies along with several others led to limited access to vital services for immigrants and caused additional strain in the healthcare system (Edward, 2014).

State governments have also enacted policies in response to federal policies, creating a layered, jurisdictional patchwork. California prohibits local jurisdictions from mandating the use of E-Verify and extends protections to domestic and agricultural workers regardless of legal status (California Immigrant Policy Center, 2021) and uses state funds to expand Medicaid (California’s Medicaid program is Medi-Cal) to non-citizens ineligible under federal law (National immigration Law Center, 2021).

Our framework also includes the interplay between structure and human agency (Giddens, 1979), incorporating the theory of bounded agency. Agency is individuals’ ability to respond to changing contexts over time (Emirbayer and Mische, 1998; Mead, 1932). ‘Bounded’ agency refers to agency that is socially situated and shaped but not wholly determined by contexts; rather, individuals may creatively move within or alter their social environments (Evans, 2007). For example, when constrained by legal status, individuals can access other resources that are not contingent upon legal status or seek out opportunities to change their legal status. Legal status is neither fixed nor permanent; rather it can change by area of residence, time period, and policy (Flores and Schachter, 2019). Employment may also be an area where immigrants have a degree of agency; changing or making choices about jobs can be an important act of agency when they cannot directly change their status through immigration law. The theory of bounded agency provides a framework to understand how and why individuals act in relation to their contexts, including their subjective perceptions of the institutions they navigate and imagined future possibilities.

This integrated framework guides our analysis of immigrants’ experiences of exclusion in healthcare (which we conceptualize as a key facet of social inclusion). We investigate how immigrants’ healthcare access is shaped by institutions and their rules within three intersecting institutional domains of interest: immigration, employment and healthcare. We explore how participants accorded meaning to navigating these institutions as an immigrant, including how they perceive and interpret experiences of social inclusion or exclusion. We also seek to understand their responses to the constraints and opportunities in immigration, employment, and healthcare domains and the subsequent long-term influence on immigrants’ life trajectories in the US.

## 3. Methods

### 3.1. Study participants and recruitment

The Research on Immigrant Health and State Policy (RIGHTS) Study examines the lived experiences of Latinx and Asian immigrants in California across healthcare, employment, social services, law enforcement and education sectors. In this paper, we present findings from the qualitative portion of the study, which involved semi-structured, in-depth interviews conducted to qualitatively examine the influence of state policies and immigration status on participants’ experiences accessing healthcare. All study materials and procedures were approved by the institutional review board at UCLA (IRB#17–001352). Informed consent was obtained verbally (and audio-recorded) from all respondents before participation.

We sampled from two distinct ethnic immigrant populations to understand how experiences of policies were similar across ethnicities, not to compare experiences by ethnicity. We used a purposive sampling strategy based on ethnic and geographical criteria. First, we focused on a single group from each of California’s large and heterogeneous Asian and Latinx immigrant populations: Chinese and Mexican. This allowed us to examine experiences for populations that 1) share a long history of migration to the US and California, including traditionally migrating for economic reasons, and experiencing exclusion by federal immigration law (Lee, 2002) and 2) have both experienced racialized exclusion (e.g. perceived as a threat to dominant culture or labor competition). Additionally, these groups include a diversity of legal statuses. Second, we sampled participants from one county with a high and one with a low “warmth of welcome,” Los Angeles and Orange, respectively (Pastor et al., 2012).

Participants were eligible if born in Mexico or China (including Hong Kong and Taiwan). Participants were identified through research team networks, community partner referrals (e.g., community organizations, religious organizations), and snowball recruitment.

In-depth, one-on-one semi-structured interviews were conducted from August 2018–August 2019 by 9 trained bilingual interviewers. Respondents used pseudonyms to maintain confidentiality. Interviews were conducted approximately 1-h in English, Spanish, Mandarin or Cantonese. Respondents were asked to describe their experiences as immigrants in the US (e.g., “What have been your challenges as an immigrant?“) and navigating the healthcare system (e.g., “Tell me about an experience you had when you got healthcare.“). Employment experiences emerged naturally from participants’ descriptions of their broader challenges in the US. Interviewers probed about experiences and processes related to immigration, employment, and healthcare, resulting in descriptions of how experiences unfolded. Interviews were audio recorded and transcribed verbatim.

### 3.2. Data analyses

Transcripts were analyzed in Dedoose, Version 8.0.035 (Los Angeles, CA: SocioCultural Research Consultants, LLC). Mandarin, Cantonese, and a portion of Spanish (20%) interviews were translated directly into English and coded with interpreter notes to maintain the precision and accuracy of the original language, while explaining cultural expressions and idioms. Because we had limited Chinese-speaking members, time, and funding, we focused resources towards translating Chinese interviews into English. Bilingual Spanish-English team members were able to apply English codes to Spanish transcripts and translate specific excerpts for further analysis. The codebook was developed by the research team (including the interviewers) which began during the process of conducting interviews, following a constructivist grounded theory approach. Constructivist grounded theory recognizes the role of researcher as author, seeks to identify and account for the temporal, cultural, and structural contexts that may influence and further explain participant experiences and assist in their interpretation (Merriam, 2009).

To ensure the trustworthiness of any team member’s coding decisions or interpretations of the data, the research team met weekly to discuss and reconcile such differences where they emerged, documenting all changes. Through this iterative process, the codebook grew to 175 unique codes under broad categories such as immigration system (e.g., legal status identity, status change), policy sectors (e.g., improved work opportunities), comparative codes (e.g., now/then), etc. Because multiple raters coded in different languages, we assessed inter-coder agreement to provide additional confidence in the credibility of the coding process. Interrater reliability tests were performed in Dedoose using a subset of 20 most-commonly used codes. Pooled Kappas of raters ranged from 0.80 to 0.96, indicating very good agreement (Landis and Koch, 1977).

Throughout the process of data collection and coding, the research team wrote memos to identify patterns and emergent themes and practice reflexivity. Because we identified experiences of exclusion in employment as a theme and were interested in experiences at the intersection of policy domains, we conducted a focused analysis examining excerpts coded with more than one policy domain related to employment, immigration, and healthcare. Themes and relationships between codes were identified through constant comparison analysis. Since “legal status change” emerged as a theme, findings were represented through the construction of matrices that stratified related excerpts by legal status. We also explored how respondents’ experiences were ordered chronologically, comparing excerpts before/after changes in legal status. Comparing legal status change incidents allowed us to integrate categories and their properties within and across individuals.

## 4. Results

Table 1 presents the characteristics of the 60 total study respondents, including 32 Mexican and 28 Chinese participants. At the time of interview, the majority of Mexican participants (59%) were undocumented, and most Chinese participants were citizens or legal permanent residents (LPR) (86%). Thirty-eight percent of Mexican and 75% of Chinese respondents described a change in their legal status. Half (n = 30) were ever undocumented (88% Mexican, 7% Chinese) and more than one-third (34%) of Mexican and over half (54%) of Chinese respondents ever held a temporary status, such as student or work visas, U-Visa, or Deferred Action for Childhood Arrivals (DACA). Most respondents were employed (68%), with 19% of Mexican and no Chinese participants reporting being unemployed, and 9% of Mexican and 36% of Chinese respondents reporting not being in the labor force. Regarding health insurance coverage, most Mexican participants were uninsured (72%), and most Chinese participants were insured (96%) at the time of interview.

### 4.1. Theme I: pathways of social exclusion and access

#### 4.1.1. Respondents’ legal status shaped their employment trajectories, which often shaped social and health insurance exclusion

Among participants who were ever undocumented, social exclusion related to labor emerged as a dominant theme. Every participant who had ever been undocumented described being denied numerous jobs because of the lack of work authorization, several using the phrase “closed doors” to describe their experiences of exclusion. Most relied on family and social networks for information or connections to informal employment opportunities. As a result, they frequently described settling for jobs that were low paying, had worse working conditions, making them susceptible to economic and physical exploitation, and lacked benefits. Several participants called themselves “people in the shadows” or felt regarded as those who “don’t count.” Undocumented participants commonly described experiences of disrespect, wage theft, lack of security, and coercive threats. One woman described a previous job experience she held while she was undocumented, comparing the experience to slavery:

“There was a company where many girls had sex with supervisors. I don’t know if it was loneliness, or it was wanting to continue having the job ... And it hurt because I feel that they didn’t like it either ... they had a padlock to go to the bathroom ... and you couldn’t go more than twice. There I understood the magnitude of slavery” (JJ, Woman, Mexican, Citizen).

Lack of legal status intersected with employment and healthcare exclusions and healthcare was thus considered unattainable: “It’s something that’s up in the sky, something unreachable, it’s unreachable to be able to have health insurance. It’s like no matter how much you try, you can’t, you can’t pay, you can’t get services” (Chibis, Woman, Mexican, Undocumented). As a result, most participants lacked expectations for obtaining employer-based insurance or using healthcare while undocumented: “We kind of just gloss over our health in general, like, that’s kind of our mindset, we don’t really think about it, because you can’t really go to a hospital” (Danny, Man, Mexican, Undocumented). While access to healthcare for undocumented immigrants can vary significantly by local jurisdiction, and some states, counties, and cities have extended health care coverage to undocumented immigrants (Jimenez, 2021; Marrow and Joseph, 2015), respondents reported only having access to emergency Medi-Cal in some circumstances (pregnancy, emergency care). Even in these cases, respondents still perceived exclusion from the healthcare system. Sarah (Woman, Chinese, DACA) described an undocumented friend’s experience with cancer, “they had access to emergency Medi-Cal because that gave them some sort of treatments because I think other than that they wouldn’t be able to access it.” Prior to having cancer, “they were basically shut out of the healthcare system entirely.” Some undocumented respondents also described experiences where managers threatened individuals with job termination if they took time off to seek healthcare, “If we need a doctor, we cannot even miss work because they put us down, ‘I am going to fire you’” (Yeni, Woman, Mexican, Undocumented). In this way, undocumented immigrants frequently experienced a ‘double jeopardy’ of healthcare access in employment; they both lacked the means to obtain insurance, lacked labor protections such as sick leave, and their health-seeking behaviors were penalized.

Despite being authorized to live and work in the US, exclusion from employment was also a theme among respondents who had ever held temporary legal status because of their need for a visa or permanent resident sponsorship. Several participants described the pressure of finding employer sponsorship and an inability to leave their employer while being sponsored, calling their experiences “frustrating” or “torturous.” This frequently excluded them from jobs they considered “mainstream,” namely, employment in larger, reputable companies with more resources that offered more benefits and opportunities to grow. One respondent described her feelings of having to “settle” for a job she did not want, but that would sponsor her work visa: “then I realized at that time that it was necessary to bow down to reality” (Sally, Woman, Chinese, LPR). Another participant described the experience of employer-based visa sponsorship as a form of bondage: “Another thing is broken is that when you are going through the process, the thing they implement here is kind of like bondage, I mean the hostage, right? (Xman, Man, Chinese, LPR)” He also expressed that sponsorship made immigrants susceptible to unfair treatment, fear of abuse, or retaliation, “you might not feel empowered to speak out or something like that.” These examples illustrate the process of bounded agency. While respondents possessed some agency to seek work, it was bounded by the structures of the employer sponsorship process.

Several participants who held employment-based visas also described intersections between employment and healthcare manifested by an inability to obtain employer-based health insurance since the only employers who would sponsor their legal status did not provide health insurance. Oftentimes, these were smaller companies with co-ethnic employers who were more sympathetic to the workers’ legal status needs (employers with less than 50 employees are not legally required to provide health insurance). Similar to the undocumented respondents, these respondents were also resigned to being uninsured. For those who sought private insurance on their own, respondents expressed challenges buying private insurance as visa-holders: “They don’t buy health insurance for us. So I needed to figure out the way to buy it on my own. It was actually quite difficult. Since I was on my OPT [Optional Practical Training] ... I was only qualified to buy the short-term insurance, like one-month. Each month it’s about $80, but nothing is covered ... You need to renew every month ... it’s really annoying, and it covers so little ... I asked them for some recommendations on insurance companies ... They said that I couldn’t because I wasn’t qualified, but I never knew why” (Mary, Woman, Chinese, H1B).” Respondents’ experiences of uncertainty and exclusion in healthcare access substantiated the uncertainty of their temporary legal status.

#### 4.1.2. Transitions to more secure or permanent legal statuses corresponded with upward trajectories in employment and healthcare

Many participants described that transitions in legal status were accompanied by subsequent changes in employment opportunities and healthcare access. JJ’s (Woman, Mexican, Citizen) experiences provide one example of how a change in legal status resulted in improved employment and healthcare. She described having several exploitative jobs while undocumented, including one in which she was not provided with adequate equipment and suffered a severe back injury while transporting goods. When she applied for worker’s compensation, her employer was antagonistic and denied and obstructed the claim. She was unable to cover ongoing healthcare expenses and faced an arduous legal dispute: “it was a long legal process ... I fell into a depression when I could not walk much anymore ... and that was like the most severe depression I had at that stage of my life.” Her undocumented status left her susceptible to multiple types of exploitation and exclusion, with little recourse to obtain needed healthcare. In contrast, after receiving legal permanent residency through her spouse, JJ more easily obtained employment, describing subsequent jobs as “calm.” She also described substantial differences in healthcare access, including the ability to receive ongoing physical therapy and mental health services: “My legal status definitely changed it ... I was able to qualify for medical insurance and up to this day I have it, and the insurance plan covers us ... Because when I had legal status, they accepted me everywhere ... there weren’t restrictions to see if I qualified for medicine, to see if a psychiatrist could see me, or so I could see a specialist.”

Numerous respondents described a similar pattern. Table 2 presents selected excerpts describing experiences in employment and healthcare before and after legal status transitions. Respondents described how attaining legal statuses that were more secure or permanent (e.g., from undocumented to Deferred Action for Childhood Arrivals (DACA), employment visas to LPR) corresponded with increased job options and improved work experiences. Many reported increased wages, receiving more benefits, the ability to change jobs, and career advancement. Participants often expressed relief after attaining more secure legal status: “they gave me the opportunity to breathe” (Anita, Woman, Mexican, DACA), and gratitude for these improved work options: “So coming from that environment and then jumping to this was, um, in a way, you feel grateful” (Danny, Man, Mexican, Undocumented).

These enhanced employment opportunities often improved respondents’ healthcare access. While many who lacked insurance before a legal status transition were able to access basic health services through free clinics or emergency Medi-Cal, they struggled to obtain ongoing, preventive, or specialized care. After transitioning to a more permanent legal status, participants commonly expressed satisfaction with obtaining healthcare, describing subsequent processes as quick, smooth, affordable, comprehensive, or superior.

### 4.2. Theme II: immigrants’ strategies to address social exclusion

#### 4.2.1. Status trade-off: participants prioritized advancing legal status

Respondents commonly reported prioritizing advancing their legal status to the detriment of their short-term employment or career opportunities and access to healthcare, demonstrating how respondents sought social inclusion while accepting the bounds of their circumstances. Many respondents viewed obtaining more secure and permanent legal statuses as a long-term solution to resolving their employment and healthcare challenges, “That way we can work ... so we can work, it’s the most important thing I always say” (Shina, Woman, Mexican, Undocumented). Some described permanent status as a way to obtain eligibility for public health insurance: “I applied for the citizenship because I wanted the social welfare here. There’s no other way because I’m broke. Also, some people only want to hire people with citizenship. If they didn’t ask for that, I wouldn’t apply for the citizenship here” (Cheung, Woman, Chinese, Citizen).

Many described settling for less desirable jobs and were either uninsured or lacked comprehensive health insurance during the lengthy legal process, often consuming years. Paradoxically, respondents also described exercising agency within employment to change legal status. One participant described reluctantly leaving what she felt was a better job because the employer was unable to sponsor her green card application when her visa was about to expire: “I needed to find another company that could apply for me ... I just felt that might be the end of the story, so I left and switched to this company” (Apple, Woman, Chinese, LPR). Another respondent described enduring an undesirable job that provided LPR sponsorship despite costs to career advancement and healthcare access; her job did not qualify as relevant work experience required for her Certified Public Accountant licensing, delaying her certification process by years, and did not offer health insurance.

Respondents justified their curtailed employment options and lack of health insurance by the prospect of permanent legal status, which could boost their long-term trajectories for employment and healthcare. One example is illustrated by Danny’s account, a former DACA recipient (also see Table 2). When Danny (Man, Mexican, Undocumented) faced expiration of his DACA status, he chose to apply for permanent residency through his spouse instead of renewing DACA. Constrained by low wages, he was unable to afford both LPR and DACA applications, and forfeiting DACA status resulted in the loss of his job and employer-sponsored insurance: “their suggestion was to apply to both of them ... and it all came down, really to money ... So I just figured I could use that money that was going to the DACA and invest into this residency that’s going to be a permanent status change instead of a temporary two-year change ... even if I’m working endless jobs or killing my back just working, at least I have that to look forward to, like knowing this is all temporary, this is all going to go away.” Recognizing the intersecting exclusions of legal status, employment, and healthcare, Danny chose to apply for LPR status. Danny’s “choice” exemplifies the bounded agency associated with his employment; although his limited earned income enabled him to apply for a status change, it failed to provide him the means to also apply for DACA renewal. Danny’s experience also demonstrates how his journey towards LPR status wove in and out of being undocumented, holding legal employment, and uninsurance. Remarkably, his trajectory towards greater social inclusion produced the variations in his healthcare access.

While these strategies were successful for some, particularly for undocumented participants who were able to change their status through marriage, or for employment visa holders who found supportive employers, many respondents lacked agency to change legal status, despite lengthy, costly, and repeated attempts. These respondents tended to be undocumented and stuck in low-wage, precarious jobs both conditions that provided little agency to direct their legal status or employment trajectories. They often expressed feeling shame and disgrace because of their failed efforts. One respondent described her feelings after an encounter with an immigration officer when, as a victim of domestic violence, she attempted to apply for a U-visa: “There is no respect for human sentiment, to our rights, our needs. Sometimes it’s paper requirements, harsh requirements like if we were an object, not like a human being, but like an object” (Chibis, Woman, Mexican, Undocumented). One DACA recipient described a disparaging experience with his employer:

“Being a supervisor, she asked me, ‘Been here so long and you have never thought about being a citizen or getting your papers?’ I really don’t know how to explain how I felt. I stared [at the supervisor] and said, ‘That story is too long to tell you, but the real truth is, it’s not your problem.’ And it feels bad to talk to a supervisor like that, but people think that we can tell the government ‘I want to be a citizen, you are going to make me a citizen today’ and the government says, ‘Oh ok, accepted.’ No, it is a very long process. It’s a lot of money. And that’s what they don’t understand because they’re born here, they never had to do any of that” (Luque, Man, Mexican, DACA recipient).

For these respondents, being undocumented was a source of self-blame, but it was their lack of agency that further marginalized them.

#### 4.2.2. Medical returns: immigrants navigated the complex bounded agency of employment to overcome barriers to healthcare

The most frequently described strategy to overcome barriers to healthcare in the US was to seek medical care in one’s country of origin, also known as “medical returns.” Medical returns reflect a particular type of intersection between legal status, employment, and healthcare. First, having legal status permitting international travel was a prerequisite for medical returns. By contrast, medical returns were not an option for undocumented respondents since they lacked legal status to leave and re-enter the US. For example, Chevo (Man, Mexican, Undocumented) consulted a doctor who recommended a medical return for needed knee surgery, “It’s going to be a lot of money ... If I were you, I’d leave, it’d be better if you left to your country.” This advice “discouraged” Chevo, and he ultimately stayed in the US.

Second, for immigrants with legal status, some faced a paradox of employment; their employment simultaneously created barriers to healthcare access yet provided means to seek medical care abroad. Yeya’s (Woman, Mexican, Citizen) work was so physically demanding that chronic work-related injuries made it impossible for her to perform her duties. She described seeking care in Mexico as her only option if she lost work and health insurance: “Well, since we can go out, we would go to Tijuana ... if we don’t have insurance ... you can go to Tijuana, right?” Her employment created the constraints and opportunities for medical returns through income earned: it was too little to afford private insurance, too much to be eligible for Medi-Cal, but enough to travel to Mexico for cheaper care. For other respondents, medical returns provided a back-up option for inadequate insurance coverage in the US. Lily (Woman, Chinese, LPR) was employed part-time and was still paying off medical debt. She enrolled in China’s public health insurance in case she needed future health services: “It is a public healthcare insurance. I told [my mom] to help me buy it ... I don’t want to pay the medical bill again.” Her employment also created the constraints and opportunities for medical returns: her employer did not sponsor insurance, her income was not enough to afford private care or cover the costs of services without insurance, yet it was enough to travel to China for care.

Chiou (Man, Chinese, LPR) immigrated to the United States at the end of his career and was a retiree. To obtain healthcare, he and his wife devised a complicated strategy that entailed alternately living in Taiwan and the US for 6-month periods, relying on Taiwan’s health system and Taiwan-based travel insurance. “Now we know how to plan it out. If we see a doctor here, we just need to file an insurance claim after we go back to Taiwan ... Every six months, since the travel insurance in Taiwan only covers 6 months. If we got sick after the six months, what could we do?”

Chiou also lamented, “The two of us return 20 times is enough, it requires so much money! ... Because I have been stuck in the problem of healthcare, I did not apply for citizenship. I have no choice ... This is equal to waiting for death, ha! It is very miserable.” Chiou’s account highlights another way employment is an integral component needed for healthcare access in the US context. In his case, past employment history constrained healthcare access because it was performed outside of the US (making him ineligible for subsidized Medicare) and provided him with too much savings to qualify for Medi-Cal but too little to afford Medicare premiums–issues which permanent residency (or citizenship) could not resolve. In other words, he was no longer able to modify his trajectory and lacked means to gain access to US healthcare, save for Taiwan’s travel insurance. While returning to Taiwan offered some access to healthcare, the process was ultimately overwhelming and unsustainable; this permanent barrier to healthcare will result in his full exclusion from life as an immigrant in the US.

## 5. Discussion

Using the framework of social exclusion and bounded agency, our examination of Chinese and Mexican immigrants’ lives in the US showed that experiences of exclusions unfolded under the intersections of immigration, employment, and healthcare policy, expanding knowledge of how immigrants navigate multi-level exclusions to obtain care. The social exclusion framework alone shows how disadvantage is produced, while the examination of agency further elucidates immigrants’ constraints and opportunities.

We found that intersections within the three spheres functioned to make Mexican and Chinese immigrants’ access to healthcare tenuous, not simply because of exclusions specific to legal status and work conditions but because of the intersection of the two. Legal status, in particular, initiated a series of exclusions in employment and healthcare. As a result, immigrants’ access to healthcare was stratified along the lines of legal status. Employment was a key area of agency yet played a paradoxical role in creating the conditions that simultaneously limited and facilitated healthcare access. This study contributes to an understanding of the diverse, varied employment-related pathways by which legal status determines immigrants’ healthcare access and how the US healthcare system is structured to deny access to immigrants.

Our findings show that it is the trajectories of immigrants, and not necessarily their legal status or employment at any single point in time, that significantly shape their access to healthcare (Torres and De Trinidad Young, 2016). As they navigate these intersections, immigrants’ trajectories may not be linear or even upward. Immigration policies are an “axis of stratification” which determine life chances and opportunities via position in the labor market, legal protections, access to services and aid (Menjívar, 2006; Menjívar and Abrego, 2012). Because the configuration of these three policy realms holds varied, multi-level intersections, our findings add to an understanding of how policies across sectors direct immigrants’ trajectories and constitute structural violence against immigrants. Through the lens of structural violence, we see how these policy structures perpetuate inequities and harm immigrants (Farmer, 1996; Grace et al., 2018).

Our findings challenge a prevalent documented-undocumented binary that has shaped knowledge on legal status and health. Literature has focused mainly on comparing lawfully present vs. undocumented immigrants. However, this ignores the heterogeneity and varying nature of documented noncitizens, limiting understanding of how other legal statuses affect life trajectories (Cebulko, 2014). We show that non-citizens’ experiences, including those without legal status and those with temporary statuses, were defined by degrees of exclusion. Although Liang and Zhou (2016) found that undocumented workers had worse working conditions and were less likely to seek care, they failed to find an effect on self-rated health. Our findings challenge this and other studies that fail to find a gradient between immigrants’ health and their legal status (Hamilton et al., 2019), showing that immigrants’ histories of legal status and employment vulnerabilities are complex–features that are masked by cross-sectional approaches.

Consistent with the literature on legal status stratification, our findings demonstrate the very real, material consequences of immigrants’ position. They show the implications of the social exclusion created by legal status but also highlight how the lack of workplace protections among noncitizens is central to the experience of being a noncitizen. Our findings build on the extensive literature concerning the workplace vulnerabilities of noncitizens (Quesada et al., 2011), showing that these vulnerabilities exist across legal statuses and immigrant groups. The precarity that is produced at the intersection of lacking permanent legal status and concern over job stability create the conditions for exploitative workplaces for both Mexican and Chinese immigrants. Among our sample, vulnerabilities and violations of workplace rights co-occurred with exclusions from health coverage, affordable healthcare, regular access to care, paid time off and sick leave benefits. We further illustrate the implications of employment as a key area of immigrants’ agency: those with the worst jobs have the fewest opportunities to improve their well-being. It is the exclusion from employment opportunities, treatment in the workplace, and denial of healthcare that also reinforced what it meant to reside in an undocumented or temporary legal status. In other words, immigration policy creates the inequitable structures that put noncitizens at risk in the workplace, while it is the failures of employment and healthcare policy to protect noncitizen workers that lends meaning to their experiences of what it means to lack citizenship.

The prominence of legal status in our findings points to the rigidity and weight of federal immigration policies in spite of California’s many immigrant-inclusive employment and healthcare policies and add to the body of multi-sectoral, multi-level immigrant health research (Philbin et al., 2018). Moreover, we failed to find notable differences across counties, which differ by local-level immigrant-inclusive policies. De Trinidad Young et al. (2018) found that in states with more inclusive policies, Latinx and Asian/Pacific Islander citizens had lower levels of poverty. However, they also found that the poverty gap between citizens and noncitizens was larger for Latinx, positing that the potential positive impact of inclusive environments fails to translate to the most vulnerable Latinx groups. Our results support this literature and extend it to healthcare; for those with the most vulnerable legal statuses, California’s inclusive state policies were unable to effectively mitigate employment exclusions and were only able to partially mitigate healthcare exclusions (e.g., emergency Medi-Cal, worker’s compensation). These findings underscore the need for increased and accelerated pathways to citizenship for all immigrants. Representing full legal inclusion, citizenship can increase opportunities for employment, access to health insurance (public and private), and social services. Quicker immigration processing can also reduce the time immigrants are stuck in deleterious work circumstances and the risk of becoming unauthorized because of bureaucratic delays.

This study also draws attention to a stratified US healthcare system for immigrants. Participants could generally access emergency care or basic primary care, but the degree of access (including financial burden) for ongoing care for chronic conditions, mental health services, or specialty care were contingent on their legal status and employment conditions. Literature has shown that legal status and employment characteristics critically determine healthcare access for Mexican and Chinese immigrants in the US (Brown and Yu, 2009; Liang and Zhou, 2016). This study adds nuance to the barriers and opportunities in employment and the creative strategies immigrants undertake (e.g., changing status, medical returns). Findings underscore how the entwining of employment and healthcare results in unequal allocation of healthcare access across US society. Because employment is key for healthcare access in the US, healthcare is thus subject to change, susceptible to market forces, and is regarded as a commodity, rather than a right or social good (Jennings and Hanson, 1995). Our findings challenge policymakers to disentangle healthcare from legal status and employment and to ensure that immigrants can access comprehensive care.

Notably, participants without employer-based insurance did not mention using Covered California, a service connecting California residents (including qualified immigrants) with private health insurance under the Patient Protection and Affordable Care Act (ACA). Since we did not specifically ask about the use of Covered California or the ACA, we can only speculate on the reasons it was not discussed. The ACA led to major gains for LPRs in California, but coverage for non-LPR immigrants only increased modestly and gaps between non-LPRs and citizens/LPRs further widened since 2014 (Porteny et al., 2020). Also, no significant changes in healthcare utilization after the ACA were observed among Asian immigrants (Chu et al., 2021). Our results are consistent with this research and provides evidence of the persistent barriers to healthcare for non-LPRs/citizens, suggesting that participants either were not qualified for this service, were unaware of it, or were still unable to afford insurance.

While this study investigated immigrants’ experiences of intersecting institutions, we did not explore intersections with other important social institutions, such as families. Examining how immigration, employment, and healthcare policies influence the social dynamics of families and their trajectories, especially when members hold different legal statuses, is a needed area of research. We also did not explore intersections with gender in this study. Future research should explore how institutions are gendered and the different ways that immigrant men and women experience and navigate these institutional domains.

Although our study did not focus on comparing experiences by ethnicity, we acknowledge that Mexican and Chinese immigrants’ experiences likely differ because of racialization and country-specific immigration policies. While there may be important intersections of legal status and race/ethnicity, this analysis is beyond the scope of our study. However, considering that we found similar themes across two large, yet distinct ethnic groups, this suggests that the implications of policy-related exclusions may be generalizable across immigrants in the US. Moreover, because we found that inclusive state and county policies are still not enough to fully mitigate the healthcare exclusions experienced by undocumented and temporary status holders, our findings regarding these populations are potentially generalizable to their counterparts in other states. Notably, because we used an institutional and policy perspective, our findings generate insights into the sociopolitical mechanisms that produce health inequities that may extend beyond the US (Fujishiro et al., 2021). Nevertheless, the current study adds to the body of evidence that more inclusive policy environments are needed to foster and advance immigrants’ health and physical well-being.

## 6. Conclusions

Immigration law plays a central role in the lived experiences of immigrants. Legal status was a major determinant of employment exclusion, which then shaped healthcare access and exclusion for Mexican and Chinese immigrants. Immigrants strategically navigated the limits and opportunities of their legal status as a primary strategy to obtain insurance and affordable healthcare. However, the constraints of immigration, employment, and healthcare policies often left them with little recourse to obtain the healthcare they needed. Our study highlights that inclusive policymaking across the sectors of immigration, employment, and healthcare is essential in efforts to expand healthcare for immigrants. Federal policymakers should increase pathways to citizenship and disentangle immigration and employment policies that increase disparities in healthcare access. Continued efforts to expand health insurance and increase access to health services among immigrants is needed, especially for those who are undocumented or hold temporary statuses. However, these efforts may not be effective without corresponding changes in immigration or employment policies. Further, immigration, employment, and healthcare policies intersect across levels, making state and local policies that expand affordable healthcare options a critical element to addressing these intersecting exclusions. The time has come, to bring immigrants out of the shadows, to free them from the multiple exclusionary policies that bind them, and to improve their health and well-being.

## Figures and Tables

**Table 1:** Sample characteristics.

	Mexican	Chinese^a^
	Mean (SD) or n (%)	Mean (SD) or n (%)

Total	n = 32	n = 28
Age		
Mean (years)	42.2 (10.4)	42.1 (16.2)
Gender		
Women	23 (72%)	22 (79%)
Time in the US		
Mean (years)	22.9 (6.6)	15.2 (10.2)
Age at migration		
Mean (years)	19.3 (8.0)	26.9 (12.5)
Legal Status		
Citizen	6 (19%)	12 (43%)
Permanent resident (LPR)	2 (6%)	12 (43%)
Temporary status^b^	5 (16%)	4 (14%)
Undocumented	19 (59%)	–
Legal Status Transitions		
Described legal status change	12 (38%)	21 (75%)
Ever held temporary status^b^	11 (34%)	15 (54%)
Ever Undocumented	28 (88%)	2 (7%)
Language of Interview		
Cantonese/Mandarin	–	21 (75%)
English	2 (6%)	7 (25%)
Spanish	30 (94%)	–
Employment status		
Employed	23 (72%)	18 (64%)
Unemployed^c^	6 (19%)	–
Not in labor force	3 (9%)	10 (36%)
Health insurance coverage		
Insured	9 (28%)	27 (96%)
Uninsured	23 (72%)	1 (4%)

Notes.

a Includes those from Hong Kong and Taiwan.

b Temporary statuses include: DACA, H1B, L1/L2, and U-Visa.

c Unemployed includes those who were jobless, looking for work, and available for work. Those who were neither employed nor unemployed were not in the labor force.

**Table 2:** Selected excerpts regarding employment and healthcare access *before* and *after* transitions in legal status and related institutions.

	Before status change		After status change	
	Employment	Healthcare coverage	Employment	Healthcare coverage

Danny: undocumented to DACA	*[Series of jobs obtained through a temp agency]* “You’re kind of at the mercy of the agency ... You’re not really going to complain if they do something negative to you or your paycheck because you know the power they have over you . they have the power to deport you . Most immigrants know, once you get a job, you’re not gonna get sick days after the month or three months like normal jobs.”	*[No insurance coverage]* “I don’t even bother looking for healthcare honestly.”	*[Full-time legal employment at a shipping company]* “That experience was just feeling normal . I know I’m going to be working this hard, I at least feel grateful that this company’s actually going to provide benefits and help me.”	*[Employer-based insurance]* “I was enrolled in their health benefit program, I did pay into that, how anybody does with their paycheck. But again, you don’t get any of those enrollments with, like other companies that you work under the table.”
Anita: undocumented to DACA	*[Part-time employee at a fast-food restaurant]* “I, with no papers, with nothing, I started working there and I was there for five years. It was difficult ... sometimes they can’t give you what you deserve. You start with the very minimum ... there is never a raise for them to give you more.”	*[No insurance coverage]* “Back then I didn’t go to the doctor [chuckles]. I stopped going to the doctor when I turned 18 years old, and they cut my Medi-Cal. From there [chuckles] from 18 to 25, I didn’t go to the doctor because ... I didn’t know if there were going to charge me, how much they were going to charge me, if I qualified for a program, so I didn’t know anything so I would try not to go.”	*[Series of better-paying and stable jobs resulting in becoming a health clinic coordinator]* “After that when DACA started ... They gave me the opportunity to say, OK I have to do better things ... So I started looking for other jobs ... that is where they started to pay me a little bit better ... I started as a receptionist and then from there I have three years working there. And from there they gave me the opportunity to advance again. Now my title there is clinic coordinator.”	*[Employer-based insurance]* “And because I have insurance through my job ... so from there I went where I belong in urgent care, and I went and they saw me quickly.”
Flower: H-1B to LPR	*[Accountant at a small company]* “I had a very steady job, but it was not something that I could focus on my CPA certification ... But it’s just like at the time, the job I had is not something I could fulfill, I could have my certification done. So, during the time, I was so frustrated.”	*[No insurance coverage]* “Before I got my green card, I don’t think I have health [insurance]. During that time, the company doesn’t provide.”	*[Employee in a public accounting firm]* “That’s the kind of career I want to pursue, and that’s the kind of focusing that I like ... Later on, I got a mainstream job, it’s very diverse, the environment is very diverse. You really feel like you can interact better.”	*[Employer-based insurance]* “Employers’ healthcare is the best.”
Mandy: Student (to LPR) to Citizen	*[Part-time employee at a small company]* “When I started working, I actually used another person’s name because I’m not supposed to work. However, you can’t live, so it was like that, you need to, as an international student, but you have no way to get the financial aid. Then you just have to work, but you’re not supposed to work legally. ”	*[Limited insurance coverage]* “Not being a citizen is that you don’t have pay for a lot of insurance.”	*[Administrator at an organization]* “Now after becoming a citizen, I feel better. Why? Maybe I’m older and make more [money] ... It’s really much better than before, and there is not much struggle. ”	*[On spouse s employer-based insurance]* “Then everything was smooth.” “It was a really good experience I would say, because they took care of everything.”
				
*Institutions Levels*		Immigration	Employment	Healthcare
			
Federal		US Citizenship and Immigration Services (*USCIS*) • Immigration and Customs Enforcement (ICE) • DACA	• Department of Labor ∘ Wage and Hour Division	• Medicaid
State		• CA Values Act (SB 54)	• Labor Commissioner’s Office • Certified Public Accountant Licensing	• Department of Healthcare Services • Medi-Cal
Local/Community		• Law enforcement agencies	• Companies/organizations • Employer policies/practices • Temp agencies	• Hospitals, Clinics • Healthcare providers • Private/Employer-sponsored insurance
